# A one-dimensional inorganic–organic hybrid compound: *catena*-poly[ethyl­enediammonium [indate(III)-di-μ-hydrogenphosphato(V)-μ-hydroxido] monohydrate]

**DOI:** 10.1107/S160053681003240X

**Published:** 2010-08-28

**Authors:** Abderrazzak Assani, Mohamed Saadi, Lahcen El Ammari

**Affiliations:** a Laboratoire de Chimie du Solide Appliquée, Faculté des Sciences, Université Mohammed V-Agdal, Avenue Ibn Battouta, BP 1014, Rabat, Morocco

## Abstract

The title compound, (C_2_H_10_N_2_)[In(HPO_4_)_2_(OH)]·H_2_O, was synthesized under hydro­thermal conditions. The structure of this hybrid compound consists of isolated inorganic chains with composition _∞_[In(HPO_4_)_4/2_(OH)_2/2_] running along [010]. The coordination of the In^III^ atom is distorted octa­hedral. The ethyl­enediammonium cation and the disordered water mol­ecule (site-occupation factors = 0.7:0.3) ensure the cohesion of the structure *via* N—H⋯O and O—H⋯O hydrogen bonds.

## Related literature

For properties of and background to indium phosphates, see: Forster & Cheetham (2003 ▶); Chen, Liu *et al.* (2006 ▶); Chen *et al.* (2007 ▶); Huang *et al.* (2010 ▶); Thirumurugan & Srinivasan (2003 ▶). For compounds with related structures, see: Chen, Yi *et al.* (2006 ▶); Li *et al.* (2006 ▶); Du *et al.* (2004 ▶). For background to bond-valence analysis, see: Brown & Altermatt (1985 ▶).
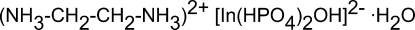


## Experimental

### 

#### Crystal data

(C_2_H_10_N_2_)[In(HPO_4_)_2_(OH)]·H_2_O
*M* *_r_* = 403.92
Monoclinic, 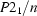

*a* = 10.0702 (3) Å
*b* = 7.4896 (2) Å
*c* = 15.6007 (5) Å
β = 99.000 (1)°
*V* = 1162.15 (6) Å^3^
*Z* = 4
Mo *K*α radiation
μ = 2.36 mm^−1^
*T* = 296 K
0.20 × 0.06 × 0.03 mm

#### Data collection

Bruker X8 APEXII CCD area-detector diffractometer
Absorption correction: multi-scan (*SADABS*; Bruker, 2005 ▶) *T*_min_ = 0.844, *T*_max_ = 0.932
13591 measured reflections
2771 independent reflections
2168 reflections with *I* > 2σ(*I*)
*R*_int_ = 0.045

#### Refinement

*R*[*F* ^2^ > 2σ(*F* ^2^)] = 0.026
*wR*(*F* ^2^) = 0.067
*S* = 1.03
2771 reflections
162 parameters
1 restraint
H atoms treated by a mixture of independent and constrained refinement
Δρ_max_ = 0.59 e Å^−3^
Δρ_min_ = −0.65 e Å^−3^

### 

Data collection: *APEX2* (Bruker, 2005 ▶); cell refinement: *SAINT* (Bruker, 2005 ▶); data reduction: *SAINT*; program(s) used to solve structure: *SHELXS97* (Sheldrick, 2008 ▶); program(s) used to refine structure: *SHELXL97* (Sheldrick, 2008 ▶); molecular graphics: *ORTEP-3 for Windows* (Farrugia, 1997 ▶) and *DIAMOND* (Brandenburg, 2006 ▶); software used to prepare material for publication: *WinGX* (Farrugia, 1999 ▶).

## Supplementary Material

Crystal structure: contains datablocks I, global. DOI: 10.1107/S160053681003240X/wm2383sup1.cif

Structure factors: contains datablocks I. DOI: 10.1107/S160053681003240X/wm2383Isup2.hkl

Additional supplementary materials:  crystallographic information; 3D view; checkCIF report

## Figures and Tables

**Table 1:** Selected bond lengths (Å)

In1—O9^i^	2.089 (2)
In1—O9	2.094 (2)
In1—O2^i^	2.135 (2)
In1—O3	2.148 (2)
In1—O6^i^	2.154 (2)
In1—O7	2.154 (2)

Symmetry code: (i) 
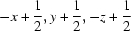
.

**Table 2:** Hydrogen-bond geometry (Å, °)

*D*—H⋯*A*	*D*—H	H⋯*A*	*D*⋯*A*	*D*—H⋯*A*
O4—H4⋯O5^ii^	0.82	1.78	2.595 (3)	174
O8—H8⋯O1	0.82	1.75	2.567 (4)	172
O9—H9⋯O10	0.86 (2)	1.93 (2)	2.780 (5)	170 (3)
O10—H10*A*⋯O1^i^	0.85	2.44	3.291 (5)	179
O10—H10*B*⋯O8^iii^	0.87	2.35	2.911 (5)	122
N1—H11*A*⋯O3^iv^	0.89	2.00	2.876 (4)	168
N1—H11*B*⋯O2^i^	0.89	2.51	3.137 (4)	128
N1—H11*B*⋯O10	0.89	2.43	3.114 (5)	133
N1—H11*C*⋯O4^v^	0.89	2.41	3.011 (4)	125
N1—H11*C*⋯O1^v^	0.89	1.98	2.823 (4)	158
N2—H22*A*⋯O5	0.89	1.87	2.750 (4)	170
N2—H22*B*⋯O6^vi^	0.89	2.06	2.911 (4)	160
N2—H22*C*⋯O7^vii^	0.89	2.05	2.892 (4)	158

Symmetry codes: (i) 
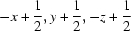
; (ii) 
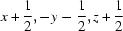
; (iii) 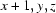; (iv) 
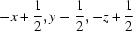
; (v) 
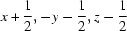
; (vi) 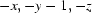; (vii) 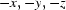.

**Fig. 1.**
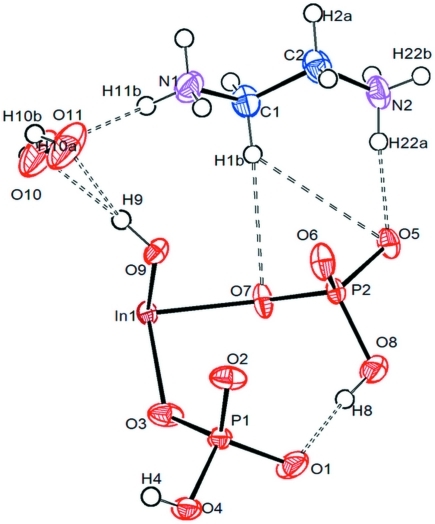
ORTEP plot of the asymmetric unit of the (H3NCH2CH2NH3)[In(HPO4)2(OH)].H2O structure. Displacement ellipsoids are drawn at the 50% probability level. Hydrogen bonds are indicated by dashed lines.

**Fig. 2.**
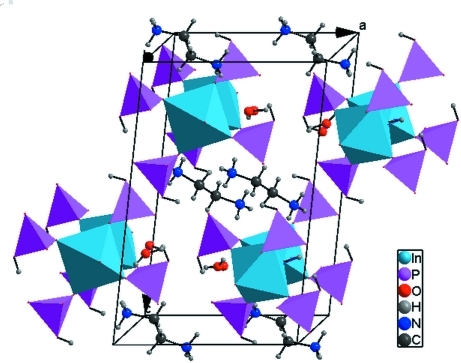
A three-dimensional polyhedral view of the crystal structure of the (H3NCH2CH2NH3)[In(HPO4)2(OH)].H2O.

**Fig. 3.**
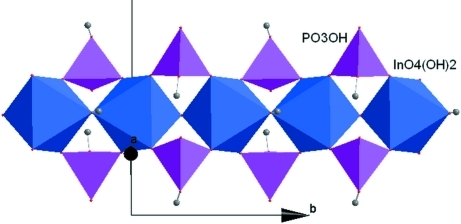
A view of an inorganic chain built up from corner sharing indium octahedra linked by HPO4 tetraedra.

## supplementary crystallographic information

### Comment

The research of new porous materials and open-framework structures in the hybrid inorganic-organic systems continues to be of great interest in the field of materials chemistry. Mainly, hybrid metal phosphates are extensively investigated due to their impressive diversity of structures which are strongly required for catalysis applications (Forster & Cheetham, 2003). Accordingly, in the past two decades, amine templated indium phosphates were in the focus of investigation, providing one-dimensional chain, two-dimensional layered and three-dimensonal open-framework structures with different In:*P* ratios (Chen *et al.* 2007; Chen, Liu *et al.* 2006; Thirumurugan & Srinivasan, 2003; Huang *et al.* 2010). In the present work, a new indium phosphate with a In:*P* ratio of 1:2, namely (H_3_NCH_2_CH_2_NH_3_)[In(HPO_4_)_2_(OH)]^.^H_2_O was hydrothermally synthesized and structurally characterized.

The asymmetric unit of the title compound is drawn in Fig. 1. A three-dimensional polyhedral view of its crystal structure is represented in Fig. 2. It shows InO_4_(OH)_2_ octahedra linked to PO_3_OH tetrahedra by sharing corners in the way to build _∞_[In(OH)_2/2_(HPO_4_)_4/2_] chains running along [010]. Fig. 3 shows the InO_6_ octahedra linked to another *via* their hydroxide vertices, giving rise to a one-dimensional linear chain. Adjacent octahedra are additionally interconnected by PO_3_OH tetrahedra by sharing their terminal O atoms with four tetrahedra. A similar connectivity is observed in the structure of (C_4_N_2_H_12_)[In_2_(HPO_4_)_2_(H_2_PO_4_)_2_F_2_] (Chen, Yi *et al.*, 2006).

The +III and +V oxidation states of the In and P atoms were confirmed by bond valence sum calculations (Brown & Altermatt, 1985). The calculated values for the two In^III+^ and P^V+^ ions are as expected, *viz*. 3.25 and 5.04, respectively. The values of the bond valence sums calculated for all oxygen atoms are: 1.33 and 1.34 for the terminal O atoms O1 and O5, 2.29, 2.30 and 2.26 for O4, O8 and O9, respectively, and 1.82 for all other O atoms except that of the water molecule (O10) which amounts to 2.12. The difference between these values is explained by the nature and the length of the P—O bonds. From the two tetrahedrally coordinated phosphorus atoms P1 and P2, each shares two O atoms with adjacent indium atoms (average distance P—O = 1.520 Å) and possesses one terminal P1═O1 = 1.510 (2) Å, P2═O5 = 1.509 (2) Å and one P1—O4H = 1.579 (2) Å, P2—O8H = 1.577 (2) Å bond. The terminal O atoms are involved in strong hydrogen bonds (see below) which likewise explains their low bond valence sum. These results are in good agreement with the framework formula and are in close agreement with those reported in the literature for similar indium phosphates (Li *et al.* 2006; Du *et al.* 2004).

The ethylenediammonum cation and the water molecules ensure the cohesion of the structure *via* N—H···O and O—H···O hydrogen bonds (Fig. 1, Table 2).

### Experimental

Single crystals of the title compound were hydrothermally synthesized from a reaction mixture of indium oxide (In_2_O_3_; 0,388 g), phosphoric acid 85%_wt_ (H_3_PO_4_; 0,35 ml), ethylenediamine (NH_2_(CH_2_)_2_NH_2_; 0,3 ml) and water (H_2_O; 10 ml). In addition, 40%_wt_ fluoric acid (HF; 0,1 ml) was added to the mixture to provide fluoride ions which can act as a mineralizing agent in the hydrothermal synthesis and can play a structure-directing role. The hydrothermal reaction was conducted in a 23 ml Teflon-lined autoclave under autogeneous pressure at 398 K for two days. The resulting product was filtered off, washed with deionized water and was dried in air. It consisted of a yellow powder in addition to a few colorless parallelepipedic crystals of the title compound.

### Refinement

All O-bound, N-bound and C-bound H atoms were initially located in a difference map and refined with O—H, N—H and C—H distance restraints of 0.82 (1) Å, 0.89 (1) Å and C–H 0.97 (1) Å, respectively. In a subsequent cycle they were refined in the riding model approximation with *U*_iso_(H) set to 1.5*U*_eq_(O) or (N) and *U*_iso_(H) set to 1.2 *U*_eq_(C). The refinement of the site occupancy of the O atoms of the water molecule shows full occupation. However, the electron density is distributed over two adjacent positions (O10 and O11). The refinement of the occupancy rates of these two positions led to a site occupancy factor of 0.7 for O10 and of 0.3 for O11, accompanied with considerable improvements in *R* and *Rw* factors.

From the synthetic conditions one might expect an incorporation of F^-^ ions. The distinction by X-ray diffraction between F^-^ and O^2-^ is difficult. However, when the relevant OH positions were replaced by F^-^, a small worsening of the reliability factors was observed. Moreover, the clearly discernible proton positions in the difference Fourier maps point to OH rather than to F. Nevertheless, the existence of a very small amount of F^-^ incorporated in the structure cannot be excluded.

### Crystal data

**Table:**

(C_2_H_10_N_2_)[In(HPO_4_)_2_(OH)]·H_2_O	*F*(000) = 800
*M**_r_* = 403.92	*D*_x_ = 2.309 Mg m^−^^3^
Monoclinic, *P*2_1_/*n*	Mo *K*α radiation, λ = 0.71073 Å
Hall symbol: -P 2yn	Cell parameters from 2771 reflections
*a* = 10.0702 (3) Å	θ = 2.6–27.9°
*b* = 7.4896 (2) Å	µ = 2.36 mm^−^^1^
*c* = 15.6007 (5) Å	*T* = 296 K
β = 99.000 (1)°	Plate, colourless
*V* = 1162.15 (6) Å^3^	0.20 × 0.06 × 0.03 mm
*Z* = 4	

### Data collection

**Table:**

Bruker X8 APEXII CCD area-detector diffractometer	2771 independent reflections
Radiation source: fine-focus sealed tube	2168 reflections with *I* > 2σ(*I*)
graphite	*R*_int_ = 0.045
φ and ω scans	θ_max_ = 27.9°, θ_min_ = 2.6°
Absorption correction: multi-scan (*SADABS*; Bruker, 2005)	*h* = −13→13
*T*_min_ = 0.844, *T*_max_ = 0.932	*k* = −9→9
13591 measured reflections	*l* = −20→19

### Refinement

**Table:**

Refinement on *F*^2^	Primary atom site location: structure-invariant direct methods
Least-squares matrix: full	Secondary atom site location: difference Fourier map
*R*[*F*^2^ > 2σ(*F*^2^)] = 0.026	Hydrogen site location: inferred from neighbouring sites
*wR*(*F*^2^) = 0.067	H atoms treated by a mixture of independent and constrained refinement
*S* = 1.03	*w* = 1/[σ^2^(*F*_o_^2^) + (0.031*P*)^2^ + 0.4367*P*] where *P* = (*F*_o_^2^ + 2*F*_c_^2^)/3
2771 reflections	(Δ/σ)_max_ = 0.001
162 parameters	Δρ_max_ = 0.59 e Å^−^^3^
1 restraint	Δρ_min_ = −0.65 e Å^−^^3^

### Special details

**Table:**

Geometry. All e.s.d.'s (except the e.s.d. in the dihedral angle between two l.s. planes) are estimated using the full covariance matrix. The cell e.s.d.'s are taken into account individually in the estimation of e.s.d.'s in distances, angles and torsion angles; correlations between e.s.d.'s in cell parameters are only used when they are defined by crystal symmetry. An approximate (isotropic) treatment of cell e.s.d.'s is used for estimating e.s.d.'s involving l.s. planes.
Refinement. Refinement of *F*^2^ against ALL reflections. The weighted *R*-factor *wR* and goodness of fit *S* are based on *F*^2^, conventional *R*-factors *R* are based on *F*, with *F* set to zero for negative *F*^2^. The threshold expression of *F*^2^ > σ(*F*^2^) is used only for calculating *R*-factors(gt) *etc*. and is not relevant to the choice of reflections for refinement. *R*-factors based on *F*^2^ are statistically about twice as large as those based on *F*, and *R*- factors based on ALL data will be even larger.

### Fractional atomic coordinates and isotropic or equivalent isotropic displacement parameters (Å^2^)

**Table:**

	*x*	*y*	*z*	*U*_iso_*/*U*_eq_	Occ. (<1)
In1	0.247040 (19)	0.02859 (3)	0.250039 (14)	0.01340 (8)	
P1	0.12174 (8)	−0.22253 (11)	0.39952 (5)	0.01587 (17)	
P2	−0.02212 (8)	−0.22671 (10)	0.15538 (5)	0.01537 (17)	
O1	−0.0298 (2)	−0.2219 (4)	0.37808 (17)	0.0389 (7)	
O2	0.1861 (2)	−0.3858 (3)	0.36661 (15)	0.0271 (5)	
O3	0.1849 (2)	−0.0520 (3)	0.36984 (15)	0.0271 (6)	
O4	0.1498 (2)	−0.2250 (3)	0.50197 (14)	0.0235 (5)	
H4	0.2297	−0.2449	0.5186	0.035*	
O5	−0.0986 (2)	−0.2250 (3)	0.06416 (15)	0.0233 (5)	
O6	0.0672 (2)	−0.3905 (3)	0.17185 (15)	0.0246 (5)	
O7	0.0595 (2)	−0.0564 (3)	0.17533 (16)	0.0243 (5)	
O8	−0.1324 (2)	−0.2335 (4)	0.21683 (17)	0.0366 (7)	
H8	−0.0965	−0.2205	0.2673	0.055*	
O9	0.3367 (2)	−0.2199 (3)	0.23619 (15)	0.0174 (5)	
H9	0.4156 (15)	−0.208 (4)	0.222 (2)	0.026*	
O10	0.5844 (4)	−0.1357 (6)	0.1893 (3)	0.0634 (14)	0.70
H10A	0.5706	−0.0285	0.1724	0.095*	
H10B	0.6316	−0.2340	0.1945	0.095*	
O11	0.5872 (10)	−0.3060 (14)	0.2029 (9)	0.0634 (14)	0.30
N1	0.3639 (3)	−0.2375 (4)	0.0340 (2)	0.0309 (7)	
H11A	0.3562	−0.3278	0.0701	0.046*	
H11B	0.4083	−0.1482	0.0633	0.046*	
H11C	0.4086	−0.2738	−0.0076	0.046*	
N2	0.0128 (3)	−0.2744 (4)	−0.0842 (2)	0.0281 (7)	
H22A	−0.0222	−0.2442	−0.0372	0.042*	
H22B	−0.0317	−0.3677	−0.1098	0.042*	
H22C	0.0059	−0.1828	−0.1209	0.042*	
C1	0.2296 (3)	−0.1762 (5)	−0.0048 (2)	0.0276 (8)	
H1A	0.2376	−0.0727	−0.0410	0.033*	
H1B	0.1792	−0.1414	0.0406	0.033*	
C2	0.1563 (3)	−0.3220 (5)	−0.0585 (2)	0.0300 (8)	
H2A	0.1972	−0.3411	−0.1101	0.036*	
H2B	0.1632	−0.4322	−0.0254	0.036*	

### Atomic displacement parameters (Å^2^)

**Table:**

	*U*^11^	*U*^22^	*U*^33^	*U*^12^	*U*^13^	*U*^23^
In1	0.01468 (13)	0.00975 (12)	0.01524 (12)	−0.00045 (8)	0.00069 (8)	−0.00008 (8)
P1	0.0160 (4)	0.0180 (4)	0.0139 (4)	0.0005 (3)	0.0032 (3)	−0.0003 (3)
P2	0.0134 (4)	0.0151 (4)	0.0162 (4)	−0.0004 (3)	−0.0022 (3)	−0.0006 (3)
O1	0.0158 (12)	0.081 (2)	0.0204 (14)	0.0028 (12)	0.0031 (10)	0.0022 (13)
O2	0.0413 (14)	0.0192 (13)	0.0237 (13)	0.0033 (10)	0.0145 (11)	−0.0014 (9)
O3	0.0441 (15)	0.0177 (13)	0.0230 (14)	−0.0017 (10)	0.0159 (11)	0.0001 (9)
O4	0.0207 (12)	0.0355 (14)	0.0144 (12)	0.0046 (10)	0.0035 (9)	−0.0007 (9)
O5	0.0197 (12)	0.0277 (13)	0.0193 (12)	−0.0008 (9)	−0.0070 (9)	0.0009 (9)
O6	0.0204 (11)	0.0160 (12)	0.0337 (14)	0.0022 (9)	−0.0077 (10)	0.0014 (9)
O7	0.0203 (11)	0.0156 (12)	0.0335 (15)	−0.0041 (9)	−0.0064 (10)	−0.0034 (9)
O8	0.0180 (13)	0.068 (2)	0.0241 (14)	−0.0055 (12)	0.0042 (11)	−0.0058 (13)
O9	0.0156 (11)	0.0103 (10)	0.0279 (13)	−0.0003 (8)	0.0079 (9)	0.0003 (9)
O10	0.035 (2)	0.044 (2)	0.119 (4)	−0.003 (2)	0.037 (2)	−0.018 (3)
O11	0.035 (2)	0.044 (2)	0.119 (4)	−0.003 (2)	0.037 (2)	−0.018 (3)
N1	0.0255 (16)	0.0401 (19)	0.0276 (18)	−0.0053 (13)	0.0055 (13)	0.0052 (13)
N2	0.0241 (16)	0.0328 (17)	0.0243 (16)	−0.0039 (12)	−0.0058 (12)	−0.0003 (12)
C1	0.0253 (18)	0.0244 (19)	0.032 (2)	0.0018 (15)	0.0003 (15)	−0.0030 (15)
C2	0.0292 (19)	0.0234 (19)	0.036 (2)	0.0025 (15)	−0.0010 (16)	−0.0070 (15)

### Geometric parameters (Å, °)

**Table:**

In1—O9^i^	2.089 (2)	O9—H9	0.862 (17)
In1—O9	2.094 (2)	O10—O11	1.293 (12)
In1—O2^i^	2.135 (2)	O10—H10A	0.8496
In1—O3	2.148 (2)	O10—H10B	0.8728
In1—O6^i^	2.154 (2)	O11—H10B	0.7256
In1—O7	2.154 (2)	N1—C1	1.466 (4)
P1—O1	1.510 (3)	N1—H11A	0.8900
P1—O2	1.511 (2)	N1—H11B	0.8900
P1—O3	1.530 (2)	N1—H11C	0.8900
P1—O4	1.579 (2)	N2—C2	1.481 (4)
P2—O5	1.509 (2)	N2—H22A	0.8900
P2—O6	1.519 (2)	N2—H22B	0.8900
P2—O7	1.523 (2)	N2—H22C	0.8900
P2—O8	1.577 (3)	C1—C2	1.498 (5)
O2—In1^ii^	2.135 (2)	C1—H1A	0.9700
O4—H4	0.8200	C1—H1B	0.9700
O6—In1^ii^	2.154 (2)	C2—H2A	0.9700
O8—H8	0.8200	C2—H2B	0.9700
O9—In1^ii^	2.089 (2)		
			
O9^i^—In1—O9	178.25 (5)	P2—O8—H8	109.5
O9^i^—In1—O2^i^	90.18 (9)	In1^ii^—O9—In1	127.09 (10)
O9—In1—O2^i^	88.87 (9)	In1^ii^—O9—H9	122 (2)
O9^i^—In1—O3	89.24 (8)	In1—O9—H9	111 (2)
O9—In1—O3	91.66 (8)	O11—O10—H10A	169.0
O2^i^—In1—O3	178.07 (9)	O11—O10—H10B	32.4
O9^i^—In1—O6^i^	90.97 (8)	H10A—O10—H10B	152.4
O9—In1—O6^i^	87.60 (8)	O10—O11—H10B	40.1
O2^i^—In1—O6^i^	92.06 (9)	C1—N1—H11A	109.5
O3—In1—O6^i^	86.11 (9)	C1—N1—H11B	109.5
O9^i^—In1—O7	89.32 (8)	H11A—N1—H11B	109.5
O9—In1—O7	92.14 (8)	C1—N1—H11C	109.5
O2^i^—In1—O7	89.67 (9)	H11A—N1—H11C	109.5
O3—In1—O7	92.16 (9)	H11B—N1—H11C	109.5
O6^i^—In1—O7	178.24 (9)	C2—N2—H22A	109.5
O1—P1—O2	113.55 (15)	C2—N2—H22B	109.5
O1—P1—O3	112.56 (15)	H22A—N2—H22B	109.5
O2—P1—O3	110.60 (13)	C2—N2—H22C	109.5
O1—P1—O4	103.80 (13)	H22A—N2—H22C	109.5
O2—P1—O4	108.44 (13)	H22B—N2—H22C	109.5
O3—P1—O4	107.41 (14)	N1—C1—C2	110.2 (3)
O5—P2—O6	111.58 (13)	N1—C1—H1A	109.6
O5—P2—O7	111.43 (13)	C2—C1—H1A	109.6
O6—P2—O7	110.81 (13)	N1—C1—H1B	109.6
O5—P2—O8	105.63 (14)	C2—C1—H1B	109.6
O6—P2—O8	109.01 (15)	H1A—C1—H1B	108.1
O7—P2—O8	108.16 (14)	N2—C2—C1	110.5 (3)
P1—O2—In1^ii^	137.89 (14)	N2—C2—H2A	109.5
P1—O3—In1	133.57 (14)	C1—C2—H2A	109.5
P1—O4—H4	109.5	N2—C2—H2B	109.5
P2—O6—In1^ii^	139.63 (14)	C1—C2—H2B	109.5
P2—O7—In1	139.23 (14)	H2A—C2—H2B	108.1

Symmetry codes: (i) −*x*+1/2, *y*+1/2, −*z*+1/2; (ii) −*x*+1/2, *y*−1/2, −*z*+1/2.

### Hydrogen-bond geometry (Å, °)

**Table:**

*D*—H···*A*	*D*—H	H···*A*	*D*···*A*	*D*—H···*A*
O4—H4···O5^iii^	0.82	1.78	2.595 (3)	174
O8—H8···O1	0.82	1.75	2.567 (4)	172
O9—H9···O10	0.86 (2)	1.93 (2)	2.780 (5)	170 (3)
O10—H10A···O1^iv^	0.85	2.44	3.291 (5)	179
O10—H10B···O8^v^	0.87	2.35	2.911 (5)	122
N1—H11A···O3^ii^	0.89	2.00	2.876 (4)	168
N1—H11B···O2^iv^	0.89	2.51	3.137 (4)	128
N1—H11B···O10	0.89	2.43	3.114 (5)	133
N1—H11C···O4^vi^	0.89	2.41	3.011 (4)	125
N1—H11C···O1^vi^	0.89	1.98	2.823 (4)	158
N2—H22A···O5	0.89	1.87	2.750 (4)	170
N2—H22B···O6^vii^	0.89	2.06	2.911 (4)	160
N2—H22C···O7^viii^	0.89	2.05	2.892 (4)	158

Symmetry codes: (iii) *x*+1/2, −*y*−1/2, *z*+1/2; (iv) −*x*+1/2, *y*+1/2, −*z*+1/2; (v) *x*+1, *y*, *z*; (ii) −*x*+1/2, *y*−1/2, −*z*+1/2; (vi) *x*+1/2, −*y*−1/2, *z*−1/2; (vii) −*x*, −*y*−1, −*z*; (viii) −*x*, −*y*, −*z*.
